# Serum concentrations of 25-hydroxyvitamin D and immunoglobulins in an older Swiss cohort: results of the Senior Labor Study

**DOI:** 10.1186/1741-7015-11-176

**Published:** 2013-08-01

**Authors:** Benjamin Sakem, Cristina Nock, Zeno Stanga, Pedro Medina, Urs E Nydegger, Martin Risch, Lorenz Risch

**Affiliations:** 1 Division of Clinical Chemistry, Labormedizinisches Zentrum Dr. Risch, Waldeggstrasse 37, CH-3097, Liebefeld bei Bern, Switzerland; 2 Private Medical Office, Grundgasse 2, CH-6460, Altdorf, Switzerland; 3 Division of Endocrinology, Diabetes and Clinical Nutrition, University Hospital, Bern, Switzerland; 4 Division of General Internal Medicine, University Hospital, Bern, Switzerland; 5 Central Laboratory, Kantonsspital Graubünden, Chur, Switzerland; 6 Private University of the Principality of Liechtenstein, Triesen, Principality of Liechtenstein; 7 Division of Clinical Biochemistry Medical University Innsbruck, Innsbruck, Austria

**Keywords:** 25(OH)D, Immunoglobulins, IgA, Complement, Humoral immunity, Sunlight exposure, Older people

## Abstract

**Background:**

Vitamin D and the components of humoral immunity play important roles in human health. Older people have lower 25-hydroxyvitamin D (25(OH)D) serum levels than younger adults. We aimed to determine the levels of 25(OH)D serum concentrations in healthy senior citizens and to study their relationship to the levels of components of humoral immunity.

**Methods:**

A total of 1,470 healthy Swiss men and women, 60 years or older, were recruited for this study. A total of 179 subjects dropped out of the study because of elevated serum concentrations of C-reactive protein. Fasting blood sera were analyzed for 25(OH)D with the high-performance liquid chromatography (HPLC) and for parathyroid hormone (PTH), immunoglobulins and complement C4 and C3 concentrations with immunoassays. The percentage of participants in each of the four 25(OH)D deficiency groups - severely deficient (<10 ng/ml), deficient (10 to 20), insufficient (21 to 29 ng/ml) and normal (>=30 ng/ml) - were statistically compared. The relationship of the major components of the humoral system and age with 25(OH)D levels was also assessed.

**Results:**

About 66% of the subjects had insufficient levels of 25(OH)D. Normal levels of 25(OH)D were found in 26.1% of the subjects of which 21% were males and 30.5% were females (total study population). Severely deficient levels of 25(OH)D were found in 7.98% of the total study population. Low levels of 25(OH)D were positively associated with IgG2 (*P* = 0.01) and with C4 (*P* = 0.02), yet were inversely related to levels of IgG1 and IgA (*P* < 0.05) and C3 (*P* = 0.01). Serum levels of total IgA, IgG, IgG2 and IgG4 peaked together with 25(OH)D during late summer.

**Conclusions:**

Approximately two-thirds of the healthy, older Swiss population presented with Vitamin D insufficiency. The incremental shift in IgA and C3 levels might not necessarily reflect a deranged humoral immune defense; however, given the high prevalence of vitamin D deficiency, the importance of this condition in humoral immunity will be worth looking at more closely. This study supports the role of vitamin D in the competent immune system.

## Background

The biological role of fat-soluble vitamin D has been shown in recent studies to extend far beyond its role in calcium homeostasis and bone health [1-4]. Currently, the structure of vitamin D receptors (VDRs) and their functions are well understood. Once the receptors bind to their ligand, VDRs dimerize with an isoform of the retinoid X receptor (RXR). Many body tissues express VDR; furthermore, VDR-RXRs are found on a variety of cells, including monocytes/macrophages [3], kidney cells [5], cardiomyocytes [6], skin cells [7] and liver cells [8]. At each corresponding organ site, the vitamin D/VDR system is associated with functional organ performance.

Experimental, epidemiological and clinical studies have shown inverse associations of low vitamin D status with longevity and with retarding immunosenescence [9]. 1,25-dihydroxyvitamin D3 (1,25(OH)_2_D_3_) blocks the induction of T-helper-1 (T_H_1)-cell cytokines (for example, IFNγ) and promotes T_H_2-cell responses. This activity partly explains the decreased B-cell proliferation, plasma-cell differentiation and IgG secretion [6,9,10], which is an activity that conserves efficient immune defense (in particular anti-viral) [3]. The actions of 1,25(OH)_2_D_3_ on B cell function have been researched for many years [10] and this area has recently received interest regarding the mechanisms of action [6,11-14]; for example, a recent study described an inverse relation of serum total IgG levels with serum 25-hydroxyvitamin D (25(OH)D) [15]. Although some results remain to be confirmed, low serum 25(OH)D levels have been linked to all-cause cardiovascular, cancer and infection-related mortality and also to stroke. Furthermore, autoimmunity and some infectious agents may also cause disease because of insufficient 25(OH)D [11].

Throughout the life span, a significant proportion of humans have insufficient (<30 ng/ml) or deficient (<20 ng/ml) serum 25(OH)D levels. 25(OH)D severe deficiency may be defined as serum concentrations <10 ng/ml [16,17], deficiency as values <20 and insufficiency as 21 to 29 ng/ml, whereby values >30 ng/ml (75 nmol/L) shall reflect a sufficient concentration [2,18,19]. Regular exposures to sunlight foster the required hydroxylations to produce the bioactive compound. The first –OH attachment results in 25-hydroxyvitamin D and the second in 1,25-dihydroxyvitamin D (1,25(OH)_2_D). Humans acquire much of their vitamin D through a photosynthetic reaction in the skin with a small amount via digestive intake [20]. Those at risk for vitamin D deficiency are those with little sun exposure and/or poor dietary intake. Older people are especially at risk because aging lowers the amount of 7-dehydrocholesterol in the skin, reducing the capacity for vitamin D production. People with dark skin have a significantly lower mean serum level of 25(OH)D than those with light skin across the life-span, but dark skin people have a lower level of vitamin D-binding protein and consequently more bioactive Vitamin D [21]. The acquisition of Vitamin D may be hampered by increased melanin production, as is the case with dark skin or with increased age [22].

The European population is known for transient reductions of 10 to 20% of individual 25(OH)D serum levels measured during the winter months compared to the summer months, an observation that is particularly prominent in the older people [23].

The present study was initiated (i) to study the prevalence of insufficiency and deficiency levels in healthy older people and (ii) to assess the impact of these deficiencies on immunoglobulin and complement components C4 and C3 production.

## Methods

### Study population

Consecutive subjectively healthy, older volunteers aged 60 years and older were recruited between February 2009 and July 2010 as part of the Senior Labor Study, which is an ongoing investigation in the canton of Berne (Switzerland) initially aimed at creating appropriate reference intervals (RIs) for several analytes in older persons (http://www.seniorlabor.ch). The study participants were contacted through newspaper advertisements and different clubs and associations that had a high probability of having healthy, older members (for example, alpine clubs, sports clubs) and through the personal contacts of collaborators of the study organization.

A personal history of each subject was taken, anthropometric measurements were performed, and fasting venous blood was drawn into S-Monovette tubes (Sarstedt, Sevelen, Switzerland). Of the 1,470 participants, 179 with a high total leukocyte count (>11.5 G/L) and/or high C-reactive protein levels (>5 mg/L) were excluded from further analysis since such laboratory cutoffs are strong indicators of the presence of systemic inflammation, mostly for upper respiratory tract infections. Of the remaining 1,291 subjects, 558 were between 60 and 69 years old, 498 between 70 and 79 years old and 235 were 80 years old or older (Table 1).

**Table 1:** Percentage of healthy older subjects in the different groups of vitamin D deficiency according to gender

	**25(OH)D (ng/ml)**											
	**Men (600)**				**Women (691)**				**Total (1,291)**			
**Age groups**	**<10**	**10 to 20**	**21 to 29**	**≥30**	**<10**	**10 to 20**	**21 to 29**	**≥30**	**<10**	**10 to 20**	**21 to 29**	**≥30**
60 to 69 (558)	4.4	29.3	39.9	26.4	3.5	26.7	32.3	37.5	3.9	28.5	36.0	32.1
70 to 79 (498)	10.1	38.3	29.1	22.5	8.5	26.6	30.3	34.7	9.2	31.9	29.7	29.1
≥80 (235)	13.0	36.0	28.0	23.0	16.3	38.5	20.0	25.2	14.9	37.5	23.4	24.6
Total (1,291)	8.0	33.8	33.8	24.3	8.0	28.9	29.1	34.0	8.0	31.2	31.3	29.5

Numbers are percentages of participants classified according to 25(OH)D level group: <10 ng/ml (severe deficiency), 10 to 20 ng/ml (deficiency), 21 to 29 (insufficiency) ng/ml and ≥30 ng/ml (normal).

This study was conducted in accordance with the ethical guidelines of the 1975 Declaration of Helsinki, and informed consent was obtained from all participants. Ethical approval for the present study was obtained from the Berner Kantonal Ethics committee (KEK-Berne-Study Nr 166/08), Bern, Switzerland.

### Laboratory testing

All assays were done at our institution. For 25(OH)D concentration measurements, we used the LaChrom Elite apparatus provided by Hitachi (Hitachi Medical Systems Europe, Zug, Switzerland). 25(OH)D_2_ and 25(OH)D_3_ (derived from cholecalciferol) were distinguishable using this method; for this study, only the latter, more stable animal-origin 25(OH)D_3_ levels are reported. The US National Institute of Standards and Technology (NIST) calibration standard reference material (http://www.nist.gov) was consistently followed with each test batch, and only the values complying with the requirements were released; the inter- and intra-assay variations were <5%. The baseline total 25(OH)D levels were categorized as normal, insufficient, deficient and severely deficient using levels adapted from the definitions proposed earlier [17,24]. The IMMULITE 2000 assay (Siemens Healthcare Deutschland, Erlangen, Bavaria, Germany) was used for quantification of serum 1 to 84 parathyroid hormone (PTH); this is a solid-phase two-site chemiluminescent immunoassay with a monoclonal mouse capture antibody and a polyclonal goat signal antibody conjugated to alkaline phosphatase.

The levels of immunoglobulins, where applicable, were measured using the BN Prospec® Nephelometry System (Siemens, Zurich, Switzerland), incorporating stabilized human serum as the N Protein Standard SL. Subclass-specific sheep anti-human IgG1 and IgG2 were used in conventional nephelometry and anti-human IgG3 and IgG4 in this system is loaded on polystyrol particles to enhance sensitivity for quantitation of the cognate subclass. For C3 und C4, we used sodium azide preserved anti-C3c antibodies <6.4 mg/ml and anti-C4 <5.4 mg/ml. The company recommends application of the following reference intervals established with serum and/or plasma samples from healthy adults: C3/C3c of 0.9 to 1.8 mg/ml and C4/C4c of 0.1-0.4 mg/ml.

### Statistical procedures

The serum concentration values of 25(OH)D, immunoglobulin classes, IgG subclasses and complements were statistically analyzed using SPSS version 17.0 for Microsoft Windows (SPSS, Inc., Chicago, IL, USA). All statistical analyses were based on two-sided hypothesis tests with a significance level of *P* <0.05. The Kruskal-Wallis test was used to test for statistically significant associations of 25(OH)D and immunoglobulins in more than two groups. Further, the Mann–Whitney rank sum was used to statistically compare the levels of various immunoglobulins and complement components by categories of 25(OH)D levels (normal, insufficient, and deficiency groups). Univariate linear regression analysis was used to examine the relationship between 25(OH)D as the dependent variable and age, immunoglobulin and complement components as the independent variables.

## Results

**Figure 1:**
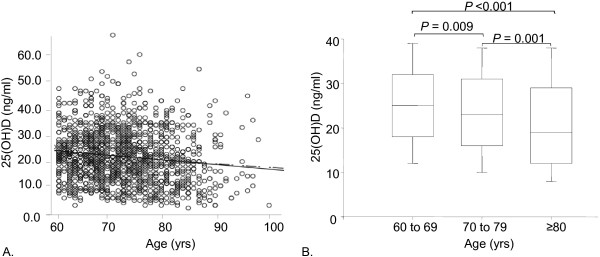
**25(OH)D levels in healthy older Swiss citizens. A**: The regression line illustrates reduction of these levels in 1,291 men and women (*P* < 0.01). **B**: The same data as in A were sorted into three age groups and are here represented in box plots. They further illustrate the decrease of 25(OH)D levels by decrements of median and interquartile ranges in relation to aging.

The entire cohort of 1,291 people studied revealed a significant correlation between aging and 25(OH)D levels (Figure 1A). To analyze in detail the effect of aging on 25(OH)D, participants were divided into three age groups (60 to 69 years, 70 to 79 years and >80 years) (Figure 1B). Only 26.1% of the subjects had normal 25(OH)D levels (>30 ng/ml). These consisted of 21% of the males and 30.5% of the female participants of the total study population. The detailed distribution of the subjects into the respective 25(OH)D deficiency groups is presented in Table 1. Severe 25(OH)D deficiencies (levels <10 ng/ml) were observed in 8.0% (M: 8.0%, F: 8.0%) of the subjects. However, it should be noted that subjects became increasingly 25(OH)D deficient with increasing age, with a prevalence of severe deficiency 3.5%, 8.5% and 16.3% in women and 4.4%, 10.1% and 13.0% in men in the progressively older age groups (Table 1). About 50% of the healthy older Swiss over 70 years have insufficient 25(OH)D levels. Serum PTH levels inversely correlated with 25(OH)D) (*P* <0.001) (Table 2). 25(OH)D levels were significantly different according to the month the samples were collected (*P* <0.001) (Additional file 1).

**Table 2:** PTH, immunoglobulins and complement C4 and C3 levels compared among four concentration ranges of 25(OH)D in 1,291 healthy older subjects

**Analyte**	**25(OH)D**					
	**<10**	**10 to 20**	**21 to 29**	**≥30**	***P*-value**	
	**N = 103**	**N = 403**	**N = 404**	**N = 381**		
					**Trend**	**Sd/Nor**^**+**^
PTH	7.87 ± 0.51*	4.78 ± 0.13	4.25 ± 0.10	3.73 ± 0.10	<0.01	<0.01
pg/ml						
IgA	2.32 ± 0.10	2.12 ± 0.05	2.22 ± 0.05	2.12 ± 0.05	0.12	0.036
mg/ml						
IgE	145.38 ± 50.1	75.45 ± 10.08	85.01 ± 13.17	70.37 ± 7.89	0.28	>0.05
kU/l						
IgM	0.88 ± 0.05	0.94 ± 0.04	0.96 ± 0.03	0.98 ± 0.03	0.09	>0.05
mg/ml						
IgG	10.63 ± 0.25	10.05 ± 0.11	10.28 ± 0.10	10.28 ± 0.12	0.24	>0.05
mg/ml						
IgG1	6.74 ± 0.21	6.16 ± 0.08	6.17 ± 0.07	6.41 ± 0.11	0.14	0.033
mg/ml						
IgG2	3.13 ± 0.15	3.23 ± 0.07	3.45 ± 0.07	3.24 ± 0.06	0.01	>0.05
mg/ml						
IgG3	0.38 ± 0.02	0.37 ± 0.01	0.38 ± 0.01	0.41 ± 0.03	0.37	>0.05
mg/ml						
IgG4	0.66 ± 0.06	0.60 ± 0.03	0.66 ± 0.03	0.59 ± 0.02	0.12	>0.05
mg/ml						
C4	0.22 ± 0.01	0.23 ± 0.00	0.23 ± 0.00	0.23 ± 0.00	0.02	0.01
mg/ml						
C3	1.11 ± 0.02	1.09 ± 0.01	1.7 ± 0.007	1.07 ± 0.01	0.03	0.01
mg/ml						

* Mean ± 1 SD.

^+^Sd/Nor: between the groups of Severely deficient and Normal serum concentrations of 25(OH)D.

Differences between groups were assessed by Kruskal-Wallis test.

**Figure 2:**
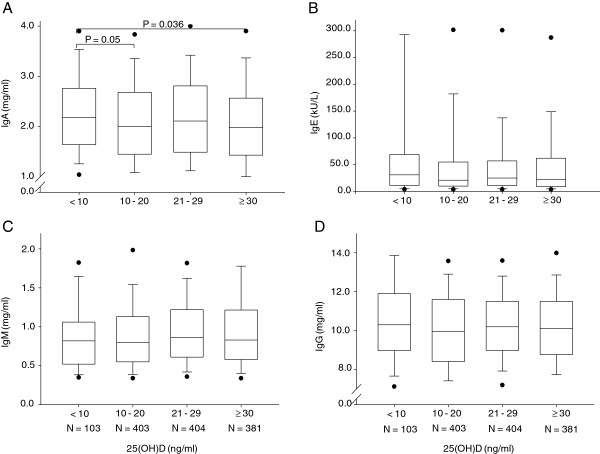
**Box and whisker plot of the relationship between immunoglobulin levels and 25(OH)D. A**: IgA levels, **B**: IgE levels, **C**: IgM levels and **D**: IgG levels, compared among the different deficiency groups of 25(OH)D in the healthy, olderSwiss population. *P*-values are indicated only when statistically significant.

We also evaluated the relationship between 25(OH)D levels and major humoral components of innate and acquired immunity. While levels of IgA inversely correlated to 25(OH)D, IgM nor of total IgG or IgE differed from changing 25(OH)D levels (Figure 2 and Table 2).

The *P*-values were obtained by either using the Kruskal Wallis analysis across the board for trend or by comparing the 25(OH)D severely deficient and the normal group.

**Figure 3:**
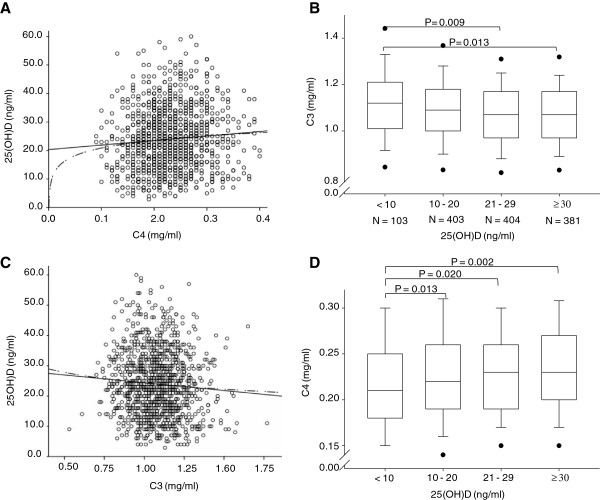
**25(OH)D levels and their relationship to C4 and C3.** Comparison of concentrations of complement components C4 and C3 in 4 different 25(OH)D serum level groups and analysis of statistical significance. **A** and **C**: Set of data points with linear regression line. **B** and **D**: Box-and-Whisker plot illustrating trend and statistical significance with variability outside the upper and lower quartiles.

The mean serum level of IgG2 significantly differed among 25(OH)D level groups (Table 2). Further, complement component C4 increased with increasing 25(OH)D (Figure 3A, B), whereas complement component C3 decreased with increasing 25(OH)D (Figure 3C, D).

Seasonal variation in immunoglobulin levels and complement components revealed differences according to the month samples were taken (Additional file 2). Significant differences were observed for total IgG (*P* = 0.001), IgG2 (*P* = 0.003), IgG4 (*P* = 0.04), complement component C4 (*P* = 0.01), and complement component C3 (*P* = 0.006). Total IgG, IgG2 and IgG4 peaked together with 25(OH)D during August.

## Discussion

This is the first time that the 25(OH)D status has been connected to humoral immunity features in healthy older subjects not receiving any real-time medical care; in fact, most studies reporting insufficient/deficient 25(OH)D levels have been performed in the context of disease, such as patients assigned to primary care [25], or having a risk for bone fractures [26], prostate cancer [27], cardiovascular disease [28], and evaluations in response to influenza or recombinant hepatitis B vaccine [29,30]. Some authors have questioned the quality of such studies, mainly for technical reasons [31]. Our study is in line with recent epidemiological investigations demonstrating a high prevalence of mild and severe 25(OH)D deficiency; in one such study, this deficiency did not significantly increase with aging [32], whereas it did in another [33].

Here, a majority (over 50%) of the older, subjectively healthy Swiss population presented with 25(OH)D insufficiency/deficiency. We observed a progressive decline in 25(OH)D levels with aging both in women and men, most likely due to lower sunlight exposure and a decline in balanced food composition for those not (yet) part of homes for retired, older persons, as those under study here. In the Osteoporotic Fractures in Men Study (MrOS) from North America, 31% of men aged 80 to 84 years had levels <20 mg/ml [33], and the proportion increased to 40% in those >85 years [33]. It can be assumed that Vitamin D insufficiency causes a substantial burden of disease (for example, falls, osteoporosis, arterial stiffness [34], cardiovascular diseases [21], autoimmune disease, infectious disease and neoplasia that would classify insufficiency as a risk factor in our subjects rather than a past event.

In conjunction with these new insights, 25(OH)D testing requests received by medical analytical laboratories are surging worldwide [35]. The present study was performed in compliance with current good laboratory practice, GLP. The cutoffs for normal, insufficient or (severely) deficient levels of vitamin D are defined at different levels based on an original proposal [36]. Currently, the deficiency decision limit of vitamin D levels is set by an international agreement at <20 ng/ml, although this cutoff is not recognized by all [18,24]. Here we considered values <10 ng/ml as indicative of severe deficiency. Since the separation into four different effectual groups cannot relate to clinical features in the healthy older persons studied, the cutoffs for 25(OH)D levels do not target risk *viz*. no risk considerations [37].

Depending on latitude and climate, seasonal/monthly variations in serum levels of 25(OH) do occur [33] or remain similar [38] and, in the cohort under study here, did vary, reflected by incremental levels towards the end of summer/fall. To the best of our knowledge, it is the first time that we here report a seasonal variation of humoral immunity components more or less consistently with 25(OH)D levels. 25(OH)D insufficiency might also occur in a sunny environment [38]. The variations in the levels of humoral immunity components through the year observed in our study might depend on changes in the microbiological mosaic affecting us with Gram positive bacteria [39] or else influenza H1N1 [40], examples of the most recently studied pathogens eliciting differential humoral immune responses. In the light of the interdependency of 25/OH)D/humoral immunity found here, Vitamin D supplementation, therefore, must be considered the whole year round on an individual risk/efficiency basis. A recent update of circannual fluctuation of 25(OH)D in 50 kidney transplant recipients found that (i) summer/winter peak-to-peak variations occur in our latitudes with an ensuing (ii) increased frequency of 25(OH)D insufficiency during winter time [41]; this finding contrasts somewhat with recent reports from Israel [38] and South Africa [42].

To establish reference intervals (RIs), different statistical procedures are commonly applied at each step. The sample size in the >80-year group of the present study exceeds the 100-observation barrier. Indeed, the significant *P*-values under non-parametric conditions highlight the findings that incremental insufficiency progresses to deficiency with increasing age, a finding not taken into account by the Endocrine Society’s Clinical Guidelines [24].

The ability of vitamin D to influence normal human immunity is highly dependent on the 25(OH)D status of individuals, and may lead to an aberrant response to infection or autoimmunity in those with insufficient vitamin D. This article describes some of the recent developments with respect to vitamin D and the immune system, and possible clinical implications. The influence that 1,25(OH) vitamin D exerts on the immune system sparks interest for its potential for reducing infections and preventing allograft rejection after transplantation [43].

Beyond the risk for rickets and osteoporosis, other diseases are increasingly being noticed for their association with Vitamin D status [44]. Therefore, we were interested in the recently suggested role of vitamin D and its active metabolite 1,25(OH)_2_D_3_ in modulating immune responses [11,45,46]. Specific cell types involved in the immune response express the enzyme CYP27B1, which produces 1,25(OH)_2_D_3_*in situ*[47]. Recent studies suggest a role of vitamin D in the reduction in the proliferation of immune cells, their maturation to plasma cells and their production of immunoglobulin [6]. Furthermore, 25(OH)D and human IgG levels have been directly linked in recent reports [15]. A potential explanation for the significant relationship among IgA, IgG2 and vitamin D discovered in the present study could be explained by the recently suggested interplay between innate and adaptive immunity and the role of T-cell cytokines and cytokine signaling [48,49], which also might explain the dependency of 25(OH)D levels and C4 and C3. In fact, interferon-γ potentiation of Toll-like receptor-induced antimicrobial peptides is associated with CYP27B1 activity that leads to enhanced bioconversion of 25D_3_ to 1,25D_3_ in the vitamin D-dependent antimicrobial pathway [50].

*In vitro*, the effects of 1,25(OH)2D3 on lymphocyte proliferation and cytokine production by PBMCs are pleiotropic [51]. Total serum IgG levels were previously reported to have a negative association with serum 25(OH)D [15], although such association was not present in cerebrospinal fluid [52] nor in our study. Low levels of 25(OH)D are thus a finding occurring in healthy older persons and might have an impact on immune defense.

## Conclusions

The majority (approximately two-thirds) of healthy older Swiss subjects present with vitamin D insufficiency. Showing the impact on serum concentrations of IgG1, IgG2, IgA and complement components C4 and C3, more so in severe cases of vitamin D deficiency, but also with insufficiency of 25(OH)D, this study supports a role of vitamin D in the immune system under immunocompetent conditions.

## Abbreviations

1: 25(OH)_2_D, 1,25-dihydroxyvitamin D; 1,25(OH)2D3): 1,25-dihydroxyvitamin D3; 25(OH)D: 25-hydroxyvitamin D; GLP: Good laboratory practice; HPLC: High-performance liquid chromatography; IFNγ: Interferon gamma; MrOS: Osteoporotic Fractures in Men Study; NIST: National Institute of Standards and Technology; PTH: Parathyroid hormone; RIs: Reference intervals; RXR: Retinoid X receptor; TH1: T-helper-1; VDRs: Vitamin D receptors.

## Competing interests

The authors declare that they have no competing interests.

## Authors’ contributions

UN, LR and MR participated in the design of the study. BS, CN, ZS, UN and LR drafted the manuscript and analyzed the data. BS, PM and CN performed the statistical analysis. All authors read and approved the final manuscript.

## Pre-publication history

The pre-publication history for this paper can be accessed here:

http://www.biomedcentral.com/1741-7015/11/176/prepub

## Supplementary Material

Additional file 1 **Monthly variation of 25(OH)D and components of the humoral immune system in older Swiss.** Monthly follow-up of mean ± 1 SD serum levels of 25(OH)D and humoral immune components. The IgE increment in summer likely reflects pollen exposure of the subjects. Number of samples collected per month are listed in brackets on the abscissa. Click here for file

Additional file 2 **Percentage increase/decrease in level of 25(OH)D and humoral immune components in older Swiss.** The percentage increase or decrease in seasonal variation of the humoral immune components showing significant correlation in seasonal variation with 25(OH)D, compared to the month of January (100%) is further illustrated here. Click here for file
