# Supplementary figures and images for: Serum concentrations of 25-hydroxyvitamin D and immunoglobulins in an older Swiss cohort: results of the Senior Labor Study
Source: BMC Med. 2013 Aug 1;11:176. doi: 10.1186/1741-7015-11-176 (PMC3751655; doi:10.1186/1741-7015-11-176)

## Slide 1
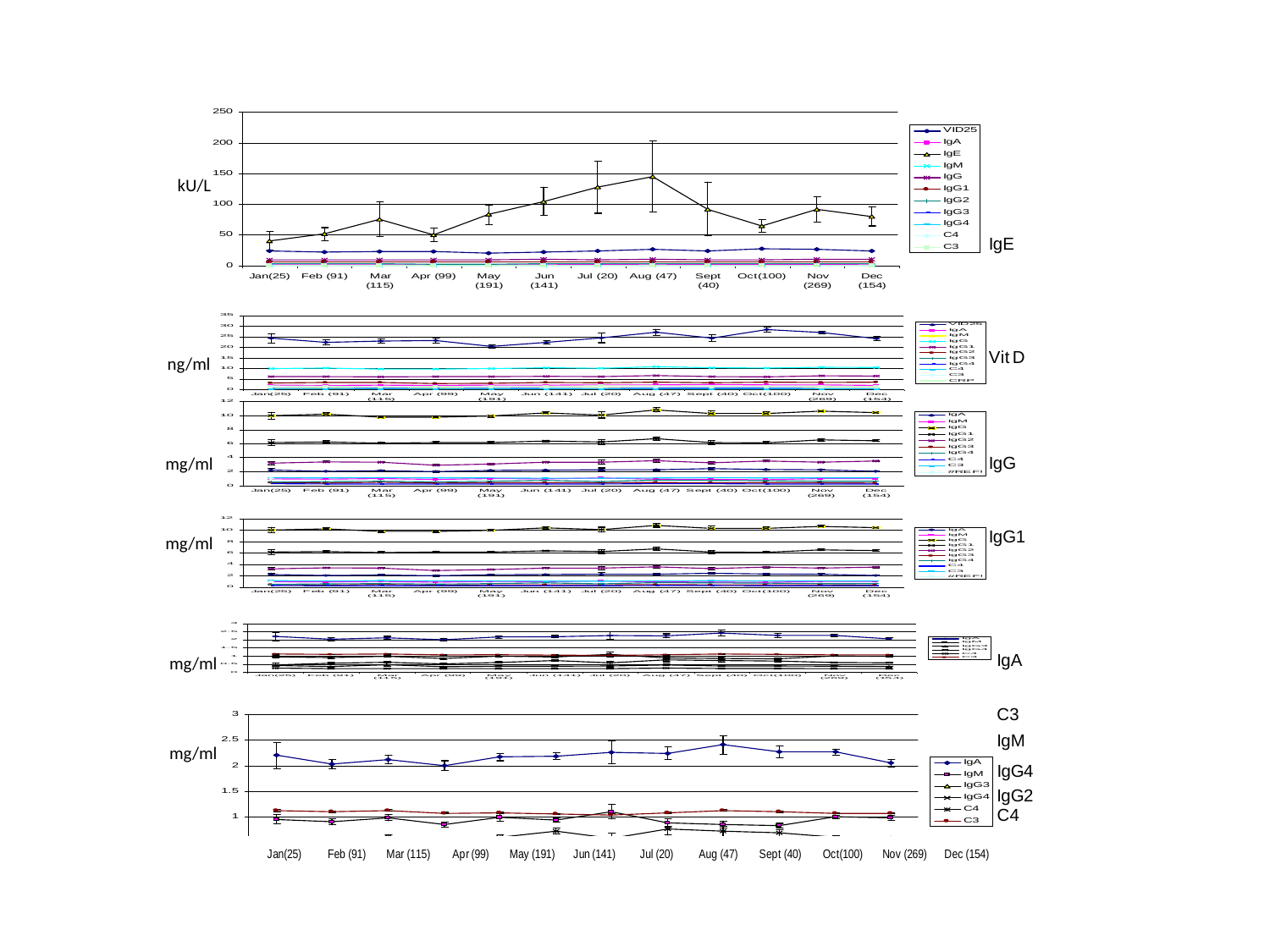

kU/L
ng/ml
 mg/ml
 mg/ml
 mg/ml
 mg/ml

Supplement: Additional file 1 — Monthly variation of 25(OH)D and components of the humoral immune system in older Swiss. Monthly follow-up of mean ± 1 SD serum levels of 25(OH)D and humoral immune components. The IgE increment in summer likely reflects pollen exposure of the subjects. Number of samples collected per month are listed in brackets on the abscissa. [file 1741-7015-11-176-S1.pptx]

## Slide 1
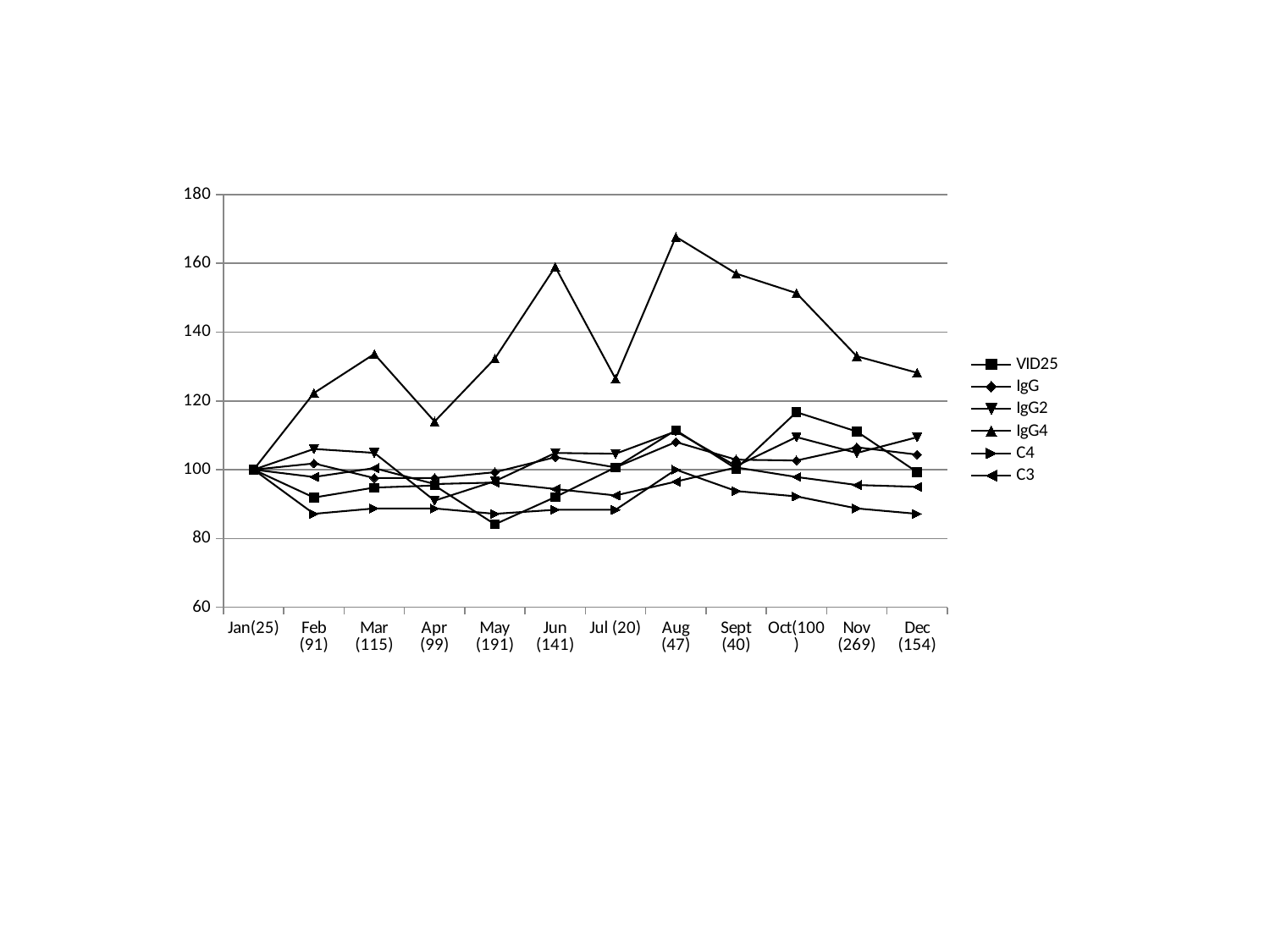

### Chart
| Category | VID25 | IgG | IgG2 | IgG4 | C4 | C3 |
|---|---|---|---|---|---|---|
| Jan(25) | 100.0 | 100.0 | 100.0 | 100.0 | 100.0 | 100.0 |
| Feb (91) | 91.89356435643565 | 101.79215058069684 | 106.00062833804589 | 122.27074235807859 | 87.15953307392996 | 97.85714285714285 |
| Mar (115) | 94.77722772277228 | 97.55706848217862 | 104.86961985548227 | 133.6244541484716 | 88.715953307393 | 100.44642857142856 |
| Apr (99) | 95.38366336633663 | 97.54705646776132 | 90.98334904178448 | 113.97379912663756 | 88.715953307393 | 95.80357142857142 |
| May (191) | 84.10478547854787 | 99.25911093311974 | 96.63839145460258 | 132.31441048034932 | 87.15953307392996 | 96.25 |
| Jun (141) | 92.02557755775578 | 103.6143372046456 | 104.83820295318881 | 158.95196506550218 | 88.32684824902724 | 94.37499999999999 |
| Jul (20) | 100.66006600660067 | 100.65078093712457 | 104.61828463713478 | 126.41921397379912 | 88.32684824902724 | 92.5 |
| Aug (47) | 111.47277227722773 | 108.0296355626752 | 111.12158341187559 | 167.68558951965065 | 100.0 | 96.51785714285714 |
| Sept (40) | 100.24752475247524 | 102.89347216659992 | 101.03675777568333 | 156.98689956331876 | 93.77431906614785 | 100.62499999999999 |
| Oct(100) | 116.70792079207921 | 102.6531838205847 | 109.4564875903236 | 151.31004366812226 | 92.21789883268482 | 97.85714285714285 |
| Nov (269) | 111.08085808580859 | 106.4977973568282 | 104.83820295318881 | 132.96943231441048 | 88.715953307393 | 95.53571428571428 |
| Dec (154) | 99.27805280528054 | 104.36523828594314 | 109.42507068803017 | 128.16593886462883 | 87.15953307392996 | 95.0 |

Supplement: Additional file 2 — Percentage increase/decrease in level of 25(OH)D and humoral immune components in older Swiss. The percentage increase or decrease in seasonal variation of the humoral immune components showing significant correlation in seasonal variation with 25(OH)D, compared to the month of January (100%) is further illustrated here. [file 1741-7015-11-176-S2.pptx]
